# Getting recovery right after neck dissection for head and neck cancer (GRRAND): trial protocol for a multicentre, pragmatic randomised controlled trial with health economic evaluation and process evaluation

**DOI:** 10.1136/bmjopen-2025-109424

**Published:** 2025-10-02

**Authors:** Chrissy Evans, Leanne Greene, Anower Hossain, Pritpal Klear, Mariam Ratna, Helen Bradley, Ranjit Lall, Hema Mistry, Seyran Naghdi, Marco Reategui, Tony Stevens, Julie Bruce, Ruth Price, Andrew Schache, Sarah Gerard Dean, Sarah Elizabeth Lamb, Toby Smith, Stuart C Winter

**Affiliations:** 1 Warwick Clinical Trials Unit, Warwick Medical School, University of Warwick, Coventry, UK; 2 Department of Health and Community Sciences, University of Exeter, Exeter, UK; 3 Oxford University Hospitals NHS Foundation Trust, Oxford, UK; 4 Liverpool University Hospitals NHS Foundation Trust, Liverpool, UK; 5 University of Liverpool Faculty of Health and Life Sciences, Liverpool, UK; 6 College of Medicine and Health, University of Exeter, Exeter, UK; 7 Institute for Applied & Translational Technologies in Surgery, University Hospitals Coventry and Warwickshire NHS Trust, Coventry, UK; 8 Nuffield Department of Surgical Sciences, University of Oxford, Oxford, UK

**Keywords:** Neck Dissection, Shoulder Dysfunction, Physiotherapy, Rehabilitation, Protocol: Randomised Controlled Trial

## Abstract

**Introduction:**

Head and neck cancer (HNC) affects the mouth, throat, salivary glands, voice box, nose or sinuses. Every year, over 12 000 people in the UK are diagnosed with HNC. Neck dissection is a key, surgical component of patient care. However, many people experience postoperative restriction in shoulder and neck movements, pain, fatigue and low mood, with only half ever returning to work.

**Methods and analysis:**

Getting Recovery Right After Neck Dissection (GRRAND) is a two-arm, multicentre, pragmatic randomised controlled trial. The trial aims to compare clinical and cost-effectiveness of a personalised physiotherapy programme (GRRAND programme) versus usual practice, National Health Service (NHS), postdischarge care.

The planned sample size is 390 participants. Participants will be recruited from across UK sites and followed up for 12 months. The primary outcome is the shoulder pain and disability index at 12 months. Economic evaluation will be conducted from a healthcare system and personal social services perspective. Secondary outcome data, including pain, function, health-related quality of life, mental well-being, health resource use and adverse events, will be collected at 6 weeks, 3, 6 and 12 months, with exercise adherence at 6 weeks. A process evaluation will determine how GRRAND is implemented, delivered and received across clinical settings, exploring what works, for whom and under what conditions. Analysis will be on an intention-to-treat basis and reported inline with the Consolidated Standards of Reporting Trials statement.

**Ethics and dissemination:**

The trial was approved by the London-Brent Research Ethics Committee (ref: 24/LO/0722) on 15 October 2024. Trial results will be disseminated via peer-reviewed publications, presentations at national and international conferences, in lay summaries and social media. This protocol adheres to the recommended Standard Protocol Items: Recommendations for Interventional Trials checklist.

**Trial registration number:**

ISRCTN13855775.

**STRENGTHS AND LIMITATIONS OF THIS STUDY**

- This will be the largest randomised controlled trial assessing a physiotherapy intervention to usual care for people after neck dissection for head and neck cancer.
- The process evaluation uses a comprehensive approach to examine implementation, delivery and contextual factors, enhancing understanding of how the Getting Recovery Right After Neck Dissection programme works in real-world settings.
- The intervention framework is flexible, offering the ability for personalised tailoring of physiotherapy, avoiding a rigid standardisation of provision.
- Capacity to deliver physiotherapy when this is not usual care may provide a barrier for some hospitals to participate.

## Introduction

 There is a global increase in head and neck cancer (HNC) incidence, attributed in part to the impact of the human papillomavirus (HPV).^1^,^3^ Annually, HNCs are diagnosed in 700 000 people worldwide, with over 12 000 new cases each year in the UK.^2^ These cancers affect the mouth, throat, salivary glands, larynx, nose and/or sinuses. The incidence of HNC has increased rapidly in the UK over the last 20 years, in particular, in the oropharynx where the role of HPV has led to a doubling in disease incidence.^4^ It is expected to increase until, at least, 2045.^5 6^ People affected by HNC are now younger and more active.^7 8^

The treatment pathway for HNC is complex due to the varied anatomical sites of disease and patient needs. While treatment may involve a single modality of surgery, radiotherapy, chemotherapy or chemo–radiotherapy, many require multimodal approaches. Neck dissection is the most common component of surgical treatment both as primary treatment and following any disease relapse. It involves surgical removal of at-risk lymph nodes in the neck.^9^ The aim of surgery is to visualise and extract lymph nodes harbouring, or at risk of harbouring, tumour.

Despite improved treatments, functional morbidity remains a long-term problem, which impacts on people’s health-related quality of life (HRQoL) and with economic societal/health service consequences. Side effects from a neck dissection can be debilitating and disabling. They include neck and shoulder problems, difficulties sleeping, fatigue and generalised anxiety.^10 11^ Postoperatively, neck dissection surgery is associated with both early and late complications. Shoulder dysfunction is by far the most common and occurs in at least half and sometimes in all patients reported in different surgical series in the literature.^12^,^15^ Over 30% of people still experience shoulder pain and reduced function 12 months postoperatively.^16^ Where shoulder and musculoskeletal pain and dysfunction appear as late complications, these can persist for 5 years and beyond.^17^ The sequelae of shoulder dysfunction and psychosocial complications are strongly associated with reduced return to work. Up to half of people report an inability to work after treatment due to shoulder disability alone.^18 19^ Psychosocial complications are also highly prevalent postoperatively, predominantly anxiety and depression, but people also experience fatigue, social isolation and body image issues.^20^

Currently, there are no national guidelines for postoperative rehabilitation after neck dissection for HNC. Physiotherapy and rehabilitation provision in the UK is not uniform.^21^ Where service provision exists in the NHS, practice is varied and includes acute respiratory care, range of motion exercises for the neck and shoulder and advice on positioning the upper limb and shoulder girdle. A booklet or leaflet may be provided to supplement this treatment.^21^ Outpatient treatment is minimal and often reactive, with patients being referred by their General Practitioner (GP) or hospital clinical team only once a problem has been identified.^22^

We previously developed and tested a structured physiotherapy programme called Getting Recovery Right After Neck Dissection (GRRAND).^23^ In this feasibility study, we randomised 36 participants undergoing a neck dissection for HNC from two NHS hospitals to receive usual NHS care versus usual NHS care *PLUS* a structured, personalised, physiotherapy-led programme (the GRRAND programme). Our feasibility study demonstrated: (1) the GRRAND programme was feasible to deliver and was acceptable to patients and NHS staff; (2) a signal with respect to improved postneck dissection shoulder/arm function, pain and HRQoL and (3) the supportive rehabilitation booklet was acceptable.^23^ While this feasibility study demonstrated that a full trial was feasible for delivery in NHS settings, clinical and cost-effectiveness had yet to be determined.

### Aims and objectives

The aim of this trial is to determine whether a personalised physiotherapy-led rehabilitation programme (the GRRAND programme) or usual practice, NHS, postdischarge care is clinically effective and cost-effective approach for improving health-related outcomes in adults after neck dissection for HNC.

## Methods and analysis

### Trial design

GRRAND is a multicentre, pragmatic randomised controlled trial with 1:1 allocation, integrated health economic evaluation and process evaluation. The trial will include a 6-month internal pilot to test detailed trial procedures, data collection and confirm the feasibility of recruitment and conduct. Participants will be followed over 12-months postrandomisation to assess shoulder function, HRQoL, adverse events (AEs) and to collect cost data for economic evaluation. The trial will be conducted in NHS hospital settings. Sites will be selected to reflect demographic and socioeconomic diversity. Priority will be given to sites serving areas with high proportions of ethnic minority populations (especially Black communities) and areas of socioeconomic deprivation, including coastal communities.

**Figure 1:**
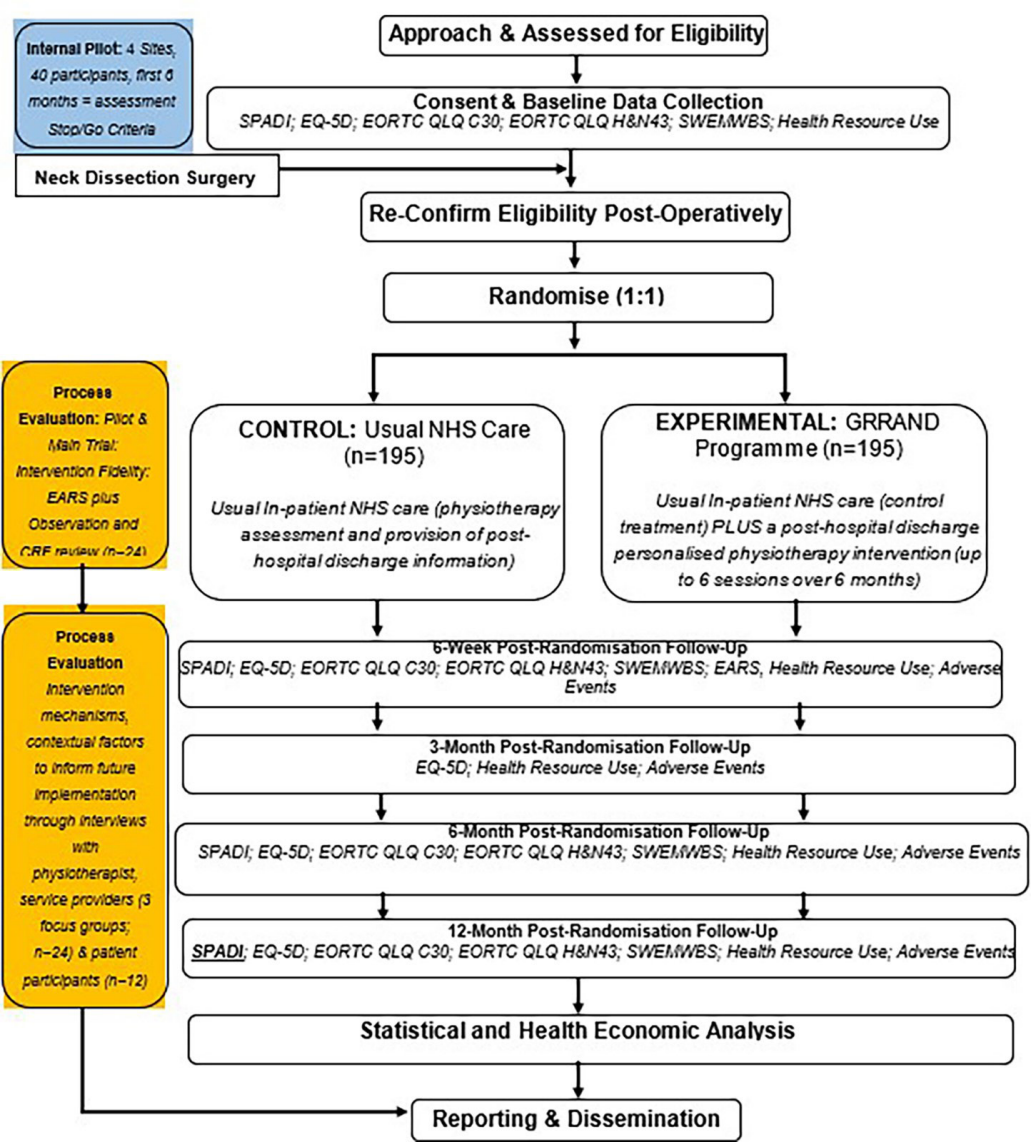
GRRAND participant flow diagram. CRF, case report forms; EARS, exercise adherence rating scale; GRRAND, getting recovery right after neck dissection; EORTC, European Organization for Research and Treatment of Cancer; SPADI, shoulder pain and disability index; SWEMWBS, short Warwick–Edinburgh mental well-being scale.

This protocol article was written following the Standard Protocol Items: Recommendations for Interventional Trials guidelines.^24^ Figure 1 is the participant flow diagram. The participant consent form is presented (online supplemental file 1). A summary of core trial information is presented in the WHO trial registration dataset (online supplemental file 2).

### Patient and public involvement (PPI)

PPI has been essential in designing the trial and will continue to be critical in its delivery and dissemination. We have collaborated with a PPI group who have had neck dissection for HNC to understand their experiences, but also to inform our trial design. Two of these PPI representatives are coapplicants on the research team. Our PPI coapplicants are integral to the Trial Management Group (TMG), attending trial management meetings, contributing to trial processes, procedures and documentation, and they will be key to our dissemination plan. A further patient partner has joined the Trial Steering Committee (TSC).

### Objectives

#### Primary objectives

- To compare the clinical effectiveness of the GRRAND programme versus usual practice, NHS, postdischarge care, on participant-reported shoulder pain and function 12 months after randomisation using the shoulder pain and disability index (SPADI).
- To compare the cost-effectiveness of the GRRAND programme against usual practice, NHS, postdischarge care from an NHS and personal social services perspective.

#### Secondary objectives

1. To evaluate postoperative pain, function, HRQoL, mental well-being, resource use and AEs at the following timepoints after randomisation based on:

- SPADI total score and pain and disability domains at 6-weeks and 6-months postrandomisation.^25^
- EORTC (European Organization for Research and Treatment of Cancer) cancer-specific questionnaires (C30 (core))^26^ and H&N35 (head and neck specific)^27^ at 6-weeks 6- and 12-months postrandomisation.
- EQ-5D-5L^28^ at 6-weeks 3-, 6- and 12-months postrandomisation.
- Exercise adherence Rating Scale (EARS)^29^ at 6-weeks postrandomisation.
- Short Warwick–Edinburgh mental well-being scale (SWEMWBS)^30^ at 6-weeks 6- and 12-months postrandomisation.
- Health resource use questionnaire at 6-weeks 3-, 6- and 12-months postrandomisation.
- AEs and postoperative complications at 6-weeks 3-, 6- and 12-months postrandomisation.

To conduct a multimethods, process evaluation to examine trial processes, implementation fidelity and contextual factors are influencing delivery and engagement. This includes assessing mechanisms of impact, such as exercise adherence using the EARS,^29^ and synthesising quantitative and qualitative data to explore what works, for whom and in what contexts.

### Outcome measures

The schedule of enrolment, interventions and assessment can be found in online supplemental file 3.

#### Primary outcome

The primary outcome is the SPADI (total score) 12-months postrandomisation.^25^ This is a 13-item participant-reported shoulder-specific instrument (0–100 and 100 worst score).^25 31^ The SPADI consists of two dimensions: pain and disability (functional activities). The pain dimension consists of five questions designed to measure the severity of an individual’s pain. The disability dimension consists of eight questions designed to measure the degree of difficulty that an individual has with various daily activities. The SPADI total score can also be obtained as a weighted average of the two domains score. It has been widely used in previous shoulder, neck and upper limb trials^32 33^ and was considered to best measure the issues that mattered most to them following a neck dissection (ie, shoulder function). It has been shown to be responsive to change in both surgical and non-surgical intervention trials.^34^

#### Secondary outcomes

Secondary outcome measures are as follows:

- SPADI total score^25^ at 6-weeks and at 6-months postrandomisation.
- SPADI total score^25^ for the pain and disability domains at 6-weeks 6-months and 12-months postrandomisation.
- EORTC QLQ-C30^26^ at 6-weeks 6-months and 12-months postrandomisation.
- EORTC QLQ-H&N35^27^ at 6 weeks, 6-months and 12-months postrandomisation.
- Mental well-being using the SWEMWBS^30^ at 6-weeks, 6-months and 12-months postrandomisation.
- Exercise adherence using the EARS^29^ at 6 weeks for both groups in addition to the last GRRAND physiotherapy appointment for those allocated to the GRRAND programme.
- EQ-5D-5L^28^ at baseline, 6-weeks, 3-months, 6-months and 12-months postrandomisation.
- Health resource use questionnaire at 6-weeks, 3-months, 6-months and 12-months postrandomisation.
  AEs and postoperative complications using the Clavien–Dindo Classification^35^ at 6-weeks, 3-months, 6-months and 12-months postrandomisation.

Process evaluation measures will include the number of staff trained, pre- and poststaff training knowledge and confidence with delivering structured HNC physiotherapy, the schedule and setting of session delivery (inpatient, outpatient or virtual), hospital and intervention case report forms (CRFs), including surgical procedure and spinal accessory nerve integrity, and observational checklists from site visits to assess fidelity.

#### Safety outcomes

AEs and serious adverse events (SAEs) related to the trial postconsent will be recorded on the appropriate CRF for return to the Warwick Clinical Trials Unit (WCTU) Team and reported to the relevant oversight committees. Further surgery, apart from the index intervention, will be considered an outcome. Treatment AE or SAE related to concomitant care, such as chemo- or radiotherapy, will not be considered recorded AEs. Persistent pain without new pathology or another event will not be considered an AE, as it will be recorded in outcome scores. SAEs will be followed up until the end of the 12-month follow-up period. All AEs and SAEs will be managed in accordance with WCTU’s standard operating procedure (SOP).

### Eligibility criteria

#### Inclusion criteria

1. People aged 18 years or over.
2. Diagnosis of HNC with requirement for a neck dissection as a part of their treatment with curative intent, Including those undergoing completion neck dissection following positive sentinel node biopsy or open neck node biopsy.
3. Able to attend outpatient physiotherapy appointments.
4. Provide informed consent.

#### Exclusion criteria

1. People for whom intensive postdischarge physiotherapy is expected (eg, scapula/scapula tip and/or latissimus dorsi free flaps or components thereof). This constitutes 3%–5% of the neck dissection population, and clinical equipoise regarding the role of physiotherapy is less evident.^21^
2. People with a pre-existing, long-term disease affecting the shoulder, for example, hemiplegia.
3. People who had prior neck dissection surgery on the affected side.
4. People undergoing only lymph node biopsy or sentinel lymph node biopsy.
5. Previous entry in the present trial.
6. Unable to adhere to trial processes.

N.B. Patients who have been excluded from the trial due to meeting exclusion criteria 4 may subsequently become eligible for inclusion *if* they require complete neck dissection.

### Participant identification, screening and withdrawals

As a part of a potential participant’s surgical multidisciplinary care outpatient appointment, and after the treatment plan has been discussed when they have been identified as requiring neck dissection, they will be approached, and if willing, screened for eligibility. Participants may also be approached in their preoperative assessment clinic or when admitted for surgery. Consent must be obtained preoperatively. Eligibility can be assessed by routine clinical evaluation, with no requirement for any specific investigation. Screening data will be entered directly on to the GRRAND trial bespoke database.

Patients who are eligible will be given verbal and written information (either in person, by post or email) about the trial and invited to discuss it further with a member of the research team. They will be given adequate time to consider participation. A delegated member of each local research team will obtain informed consent (written consent or witnessed remote verbal consent) from each participant. This will be sought preoperatively. However, following surgery, participants’ eligibility will then be verified by reviewing the medical/surgical notes. Baseline data will be collected once consent is obtained and prior to surgery.

### Randomisation and blinding

Participants will be randomly allocated to the two treatment groups via a central computer-based randomisation system provided by WCTU’s programming team, independent of the study team. This will be performed after consent has been obtained and he baseline data have been collected.

Randomisation will use a variable block size and will be 1:1 using minimisation, stratified by: age (<60 years vs>=60 years); hospital site and spinal accessory nerve sacrifice (yes/no). Randomisation will be performed by any delegated member of the local clinical or research team, using the online system.

Due to the participatory nature of rehabilitation, it is not possible to blind participants to group allocation. However, trial team members who collected or inputted data to the database will be blinded to group allocation.

### Trial treatment(s)/interventions

#### Group 1: GRRAND physiotherapy programme

A template for intervention description and replication checklist^36^ for the GRRAND intervention is included as online supplemental file 4. The development of this has been previously reported.^23^

*Delivered by:* physiotherapists trained in the principles of the GRRAND programme. Training includes background to the problem, the rationale for the trial, an overview of the GRRAND framework and trial documentation. Trained physiotherapists receive a GRRAND manual containing a detailed account of trial and intervention procedures.

*Mode of delivery:* the intervention will be personalised to the participant, allowing for flexibility for GRRAND to be delivered face-to-face in physiotherapy departments, through virtual consultations, or a hybrid of the two.

*Treatments:* participants randomised to the experimental group will receive the control group (inpatient) intervention PLUS a personalised physiotherapy-led intervention (the GRRAND programme) comprising up to six, 1-hour outpatient appointments (first session aimed for within 14 days posthospital discharge) over 6 months.

Physiotherapy treatment will include the following options.

1. *Range of motion exercises* targeting muscles and joints of the neck and shoulder impacted by neck dissection. Participants will be taught stretching and joint range of motion exercises for shoulder and neck.
2. *Progressive resistance exercises* targeting strengthening of the neck and shoulder girdle, and prevention of the onset of adhesive capsulitis. The resistance exercises will target the stabilising functions of the upper quadrant and movements of shoulder internal rotation, external rotation and abduction. Exercises will be progressed through increasingly elevated shoulder positions and the introduction of weight bearing through the upper limb. Additionally, the exercises will become increasingly ‘task specific’, targeting the specific functional goals and usual activities of daily living of the participant. Resistance loads (using resistance bands) will initially be set at a moderate level of exertion (determined by a combination of physiotherapist observation and participant-reported exertion based on the modified Borg scale of perceived exertion^37^) to permit progression, enhance motivation and adherence and reduce the possibility of symptom flare-up. Progression will be achieved by increasing the resistance load, speed and/or the number of repetitions and sets of exercises.
3. *Continued education and advice on positioning, oral health and pain management*, based on the GRRAND manual provided to participants at discharge.
4. *Education and advice on optimising exercise adherence and return to function*. This will be targeted in two ways: first, through the introduction of behaviour techniques of goal setting, pacing, behaviour modification, graded activity and for reducing fear avoidance; and second, through the discussion of barriers and facilitators to exercise and activity participation.^38^ Physiotherapists will promote independence and confidence in returning to normal activities of daily living, occupational and social pursuits and problem solving the physical neck and shoulder challenges with participants. The programme will use an established, effective approach to introduce these behaviour change techniques,^39^ developed from our previous clinical trials,^40 41^ which is within the current scope of practice for physiotherapists to deliver.
5. *Education and psychological support to address fatigue, anxiety and sleep hygiene*. Due to the high prevalence of psychological health issues postoperatively, participants will be offered strategies to support self-management of fatigue, anxiety and sleep.

*Personalised tailoring*: at the initial consultation, a physiotherapist will assess the participant to identify modifiable physical and psychosocial factors associated with poor recovery after neck dissection. The participant’s personalised GRRAND programme is based on a combination of clinical assessment and participant-identified preferences.

*How and when:* the experimental intervention will be received by participants up to six times (one assessment; five follow-up appointments) over a 6-month period to facilitate supported recovery. Reflecting the heterogeneity of clinical presentation and need, if clinically indicated, participants will be permitted to receive additional sessions over a longer duration within the trial. Nonetheless, our feasibility study results^23^ and clinical advisory group indicated that anticipated treatment should be completed within the six sessions/6-month parameters.

Reflecting normal NHS practice, the initial assessment will be 60 min as a face-to-face session. Follow-up sessions will be up to 60 min in duration, either face-to-face or virtual. The timing and mode of delivery are flexible to account for ongoing treatment, physiotherapist judgement and participant preference. Participants will be asked to perform a regular home exercise programme, for example, at least three times a week for 20–30 min.

#### Group 2: usual postoperative care

There is currently no standard treatment across the UK for rehabilitation following neck dissection for HNC.^21 42^ Accordingly, we consulted with the Clinical Advisory Group and the findings from our national survey^21^ and feasibility study^23 43^ to ensure the content and delivery of a usual practice intervention for this patient group reflects usual, NHS care.

The ‘usual care’ is based around the following four areas.

1. Advice to practise simple range of motion exercises for the face, neck and shoulder impacted by neck dissection, for the purpose of preventing the onset of postsurgical contracture and optimising the functions of swallowing and shoulder girdle movement.
2. Respiratory care, targeting the functions of sputum clearance and breathing control.
3. Mobility assessment to ensure safe hospital discharge.
4. Education on body positioning to reduce pressure and drag on the shoulder girdle, oral health to reduce food pocketing in the mouth and pain management to optimise levels of comfort and function.

As a part of ‘usual care’ prior to hospital discharge, the ward physiotherapist will provide participants with a Hospital Discharge Booklet detailing postoperative, self-management strategies inline with the standards currently used in these settings. No routine outpatient or follow-up physiotherapy will be provided as per current, best, usual NHS care.^21^

### Concomitant recovery treatments

In accordance with the pragmatic nature of this trial, participants will not be asked to desist from receiving other forms of treatment during the trial or follow-up periods. These may include contact with their GP, physiotherapist or other health professional, changes in medication or use of alternative therapies or treatments. Use of these treatments will be recorded through the health utilisation questionnaire at each follow-up period. The frequency of participants in the control group receiving physiotherapy will be reported to the oversight committees.

### End of trial

The trial will end when the analysis of 12-month follow-up data is completed. The trial will only be stopped early if mandated by the Research Ethics Committee (REC) or sponsor, following recommendations from the Data Monitoring Committee (DMC) or TSC; or if funding for the trial ceases.

### Safety reporting, AEs and SAEs

All AEs and SAEs will be defined using widely accepted standard criteria. For this trial, AEs and SAEs will be collected from the point of randomisation up to 12 months. SAEs will be reported to WCTU within 24 hours of research staff becoming aware of the event. These will be followed up until they are resolved, or until the end of the trial, and an outcome has been agreed.

### Power and sample size

Based on the feasibility study’s data,^23^ using our primary outcome of the SPADI at 12-month follow-up, to show a worthwhile difference of eight points with 90% power and 5% level of significance with an SD of 21.1,^44^ we need data on 292 participants. Allowing for 25% loss to follow-up, a total of 390 participants (195 in each group) will be required with an allocation ratio 1:1. We will require a total of 17 physiotherapists, each with an average of 12 patients to deliver the intervention. The loss to follow-up over 12 months has been modelled on 25% based on the previous literature^45^ and a greater proportion of participants in this trial being assessed at 12 month compared with our feasibility study.^23^ We will be striving to achieve a follow-up rate of >75%.

### Internal pilot

The first 6 months of randomisation will be an internal pilot, with a green target of 40 randomised. The pilot will take place in a minimum of four hospitals, selected to offer diversity across the population while also representative of centres/physiotherapists that will take part in the main trial. The progression criteria are presented in table 1.

**Table 1:** Stop–go criteria for internal pilot

Red	Amber	Green
N participants recruited	≤26 (<66%)	27–39 (66%–99%)	≥40 (100%)
Recruitment rate/site/month	< 1 participant	1–2 participants	3 participants
Number of sites opened	1 site	2–3 sites	4 sites
Intervention session attendance*†	≤27 (<70%)	28–39 (≥70%–<99%)	≥40 (100%)
Loss to follow-up†	≥10 (>25%)	1–9 (1%–25%)	0 (0%)

* Minimum one assessment and three follow-up appointments (four sessions in total) or if patient–physiotherapist goals met and agreed discharge made prior to this session number.

† Analysed at 12-month follow-up of the cohort randomised in first 6 months to obtain sufficient data.

If the internal pilot meets amber criteria, we will consult the funder, inform the TSC, review processes, look to open additional sites or amend trial processes and review again in 6 months. If the red criteria are met across all indicators, we will discuss stopping the trial with the TSC and funder.

### Statistical analysis

A detailed statistical analysis plan (SAP) will be written inline with the Estimand framework^46^ and approved by the DMC prior to the primary analysis taking place. Data will be reported inline with the CONSORT guidelines.^47^ Descriptive statistics will be constructed for baseline and follow-up data. Graphical summaries will also be created to aid the interpretation of key results.

Primary outcome analyses will adopt the Estimand framework.^46^ The SPADI score will be analysed using the treatment policy strategy (ie, intention to treat). Treatment effects (with 95% CIs) will be estimated using mixed-effect linear regression models. Both unadjusted and adjusted (for stratification variables and important patient-level covariates) estimates of the treatment effect will be presented. Secondary outcomes will be analysed using a similar approach to the primary outcome as appropriate to data and its distribution. Secondary outcomes, which are categorical, will be analysed using mixed-effect logistic regression models. Intercurrent events (ICEs) and strategies for handling ICEs: postrandomisation events that may affect the interpretation of the primary outcome would include non-adherence (including discontinuation of treatment) (ICE1). The ICE1 will be analysed using the complier average causal effect analysis.

There are no formal interim analyses. Planned subgroup analyses will be conducted. Missing data will be scrutinised and where possible, the reason for missingness recorded. If appropriate, multiple imputation will be used. Any imputation methods used for scores and other derived variables will be carefully considered and justified. In the case of missing outcome data, we will compute sensitivity analyses using imputation techniques to examine the impact of missingness. Further details on the main and sensitivity analyses will be provided in the SAP.

### Database

All data will be entered and stored in a bespoke database management system developed by the programming team at WCTU. This is supported by a detailed data management plan (DMP) produced in accordance with WCTU SOPs to ensure the high-quality data collection throughout the duration of the trial.

### Health economic evaluation

For the base-case analysis, a parallel, within-trial, economic evaluation from the NHS and personal social services perspective will be conducted, with a broader societal perspective considered in a sensitivity analysis. A health economics analysis plan (HEAP) will be developed prior to data analysis. The methods will adhere to the National Institute for Health and Care Excellence (NICE) recommended standards for economic evaluation^48^ and the internationally recognised Consolidated Health Economics Evaluation Reporting Standards guidelines for reporting economic evaluations.^49^ The cost of the intervention groups will be estimated to reflect resource inputs associated with rehabilitation and broader healthcare use. A detailed microcosting exercise for the intervention will be undertaken, including costs of development, training and supporting of physiotherapists and delivering the physiotherapy sessions. We also plan to collect information on medication, resource use in primary and community care settings (eg, GP visits, community/district nurse, physiotherapy and other allied health professional visits and home services), outpatient services (eg, Accident and Emergency (A&E) visits, physiotherapy, radiology, diagnostic imaging service, surgical and medicine services), inpatient services (eg, critical care admission and admission in hospital wards), aids and adaptation used and personal and social services (eg, meals on wheels, home care worker contacts and social worker contacts), and any broader resource use. Unit costs will be estimated from both local and national sources and reflated to current prices where necessary and presented in £ sterling. HRQoL will be measured at baseline and all follow-up timepoints using the EQ-5D-5L measure. Responses will be used to generate quality-adjusted life years (QALYs) using the appropriate value set recommended by NICE at the time of the analysis.^48^

Descriptive statistics will summarise costs and QALYs by the intervention and comparator groups. The pattern of missing data will be examined and accounted for using suitable methods for multiple imputation. Within-trial analysis using bivariate regression of costs and QALYs for the base-case analysis, with imputation of missing data will inform deterministic and probabilistic assessment of incremental cost-effectiveness and results will be expressed as an incremental cost per QALY gained. Sensitivity analyses from a societal perspective will account for productivity losses and personal expenses incurred by participants due to their condition. To measure productivity losses, we will collect information on employment status (ie, whether retired, student and full- or part-time worker), employment details (eg, employment category and employment details), educational and professional qualifications and number of days off work due to illness. Probabilistic sensitivity analyses will be undertaken to explore parameter uncertainty. Results will be graphically displayed on a cost-effectiveness plane. Cost-effectiveness acceptability curves will show the probability of cost-effectiveness at a range of willingness-to-pay thresholds. If costs and outcomes do not converge within the 12 months, economic modelling will be undertaken to explore costs and benefits over an extended time horizon. If an economic model is needed, it will be populated using data from the trial, published literature and expert opinion. A 3.5% discount rate will be applied to both costs and QALYs beyond 12 months.

### Process evaluation

This process evaluation uses a mixed-methods approach to examine the implementation, fidelity, mechanisms of impact and contextual factors influencing the delivery and uptake of the GRRAND programme.

Quantitative data will include participant adherence rates, measured using the EARS^29^ at 6 weeks for all participants and during the physiotherapy discharge session for those in the intervention group. Fidelity will be assessed through structured session observations (n~12), informal discussions with staff accompanied by field notes and CRF checks monitoring variables, such as session number and duration. Pre- and post-training questionnaires completed by physiotherapists will assess changes in confidence and knowledge. Descriptive statistics will be used to analyse quantitative data.

Qualitative data will be collected through semistructured interviews or focus groups with approximately 12 participants from the intervention group, 12 from the control group and 24 physiotherapists. Interviews will explore the acceptability of the intervention and address key themes, including contextual influences (eg, how local circumstances shaped experiences), mechanisms of change (eg, how the intervention supported recovery) and perceived outcomes. Physiotherapists will additionally reflect on implementation challenges and enablers, including local organisational conditions and culture. Purposeful sampling will be used, aiming for diversity across clinical and demographic variables, such as setting (urban vs remote), cancer stage, presence or absence of accessory nerve sacrifice, age, gender and exercise adherence levels. Sampling will also consider whether patients accessed external physiotherapy services. Qualitative data will be analysed using an interpretive descriptive approach.

**Figure 2:**
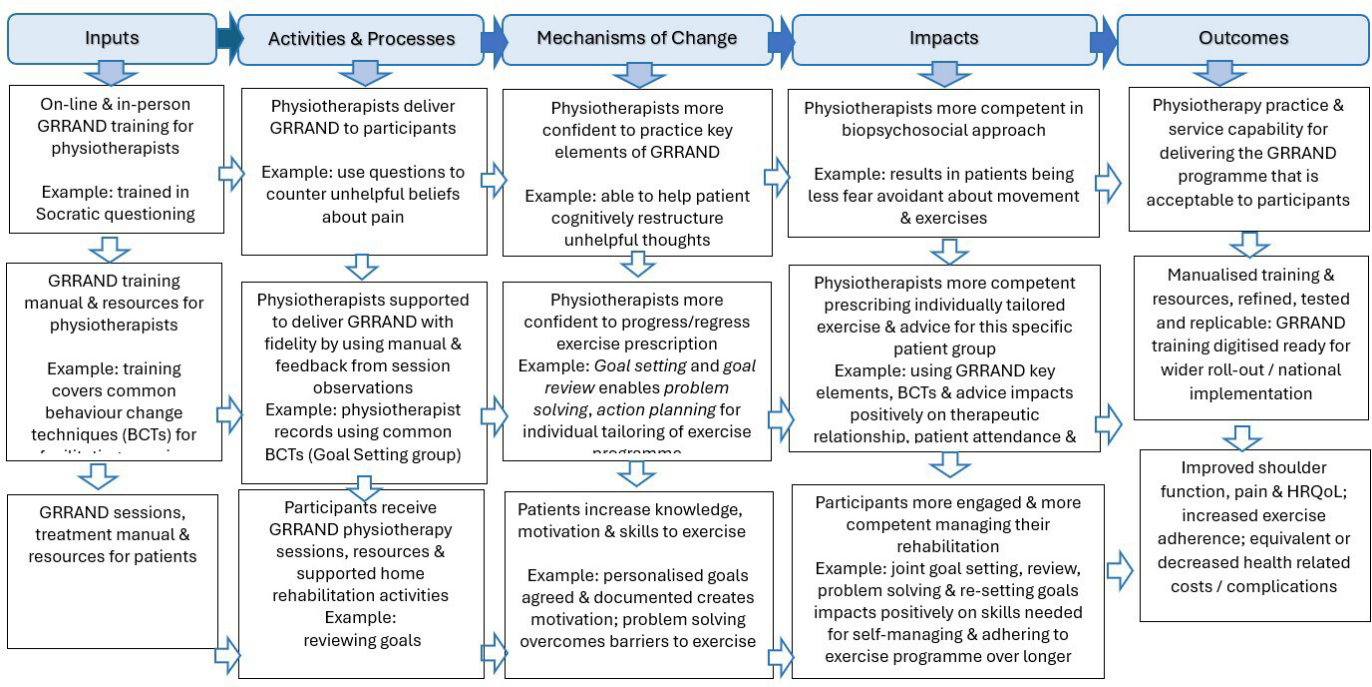
GRRAND programme logic model. GRRAND, getting recovery right after neck dissection; HRQoL, health-related quality of life.

Integration of qualitative and quantitative findings will be conducted through triangulation, using a convergence matrix to synthesise insights. This will enable the identification of patterns linking process metrics with outcomes and support understanding of what works, for whom and under what circumstances. A logic model outlining the theoretical underpinnings and intended mechanisms of the GRRAND programme is provided (figure 2) to guide and support the process evaluation.

## Ethics and dissemination

The trial was approved by the London-Brent REC (ref: 24/LO/0722). The GRRAND trial will adhere to the Declaration of Helsinki^50^ and good clinical practice principles, complying with all relevant WCTU SOPs. Participants will provide informed consent before agreeing to take part. An independent DMC and TSC will provide oversight from set up to the end of the trial. Both committees will comprise independent members as per National Institute for Health and Care Research (NIHR) and WCTU SOPs. Members will sign separate committee charters. The trial sponsor will implement DMPs. Protocol amendments will be disseminated to sites by the trial coordinating team.

## Data sharing

Deidentified data underlying the trial results will be available for non-commercial use, up to 1 year after publication of the trial findings, or from metadata stored in a university repository up to 5 years without investigator support. To access trial data, third parties must complete a data-sharing agreement with the sponsor, have an ethically approved protocol in place for use of the data and agree the approved protocol with the GRRAND TMG and WCTU Data-Sharing Committee. Data may be used for commercial purposes, according to the conditions above, but will need specific agreements in place prior to access being agreed and may include a licence fee. Analyses may include individual patient data meta-analyses or other purposes as agreed with the GRRAND TMG.

Available data will include (but is not exclusive to) deidentified individual participant data, the trial protocol, SAP, HEAP, master copy of the informed consent sheets and scripts or files used to conduct trial analyses.

## Trial registration and study timelines

The trial is registered with the nternational Standard Randomized Controlled Trial Number (ISRCTN) register (ISRCTN13855775). The current version of the protocol is V.2.0, approved on 13 March 2025. The planned dates of the study are from February 2025 to December 2027.

## Dissemination and publication

Results will be shared with trial collaborators, while the main results paper is drafted by the trial team and agreed by the TSC prior to submission to a major peer-reviewed journal. Summary briefing papers, press releases and social media posts (specifically aimed at UK audiences) will be prepared for the wider community with specific input from our PPI team. These outputs will allow for the results to be disseminated across the orthopaedic and rehabilitation communities, the wider medical community, policymakers and patients and society at large in the UK, and globally. Dissemination to trial participants will follow current Health Research Authority guidelines, with summaries available on the GRRAND website and social media as appropriate.

## Supplementary material

10.1136/bmjopen-2025-109424 online supplemental file 1

## Data Availability

Data are available on reasonable request.
