# Supplementary material for: Getting recovery right after neck dissection for head and neck cancer (GRRAND): trial protocol for a multicentre, pragmatic randomised controlled trial with health economic evaluation and process evaluation
Source: BMJ Open. 2025 Oct 2;15(10):e109424. doi: 10.1136/bmjopen-2025-109424 (PMC12496047; doi:10.1136/bmjopen-2025-109424)
Supplement: online supplemental file 1 [file bmjopen-15-10-s001.docx]

**Supplementary File 1:** Participant consent form.


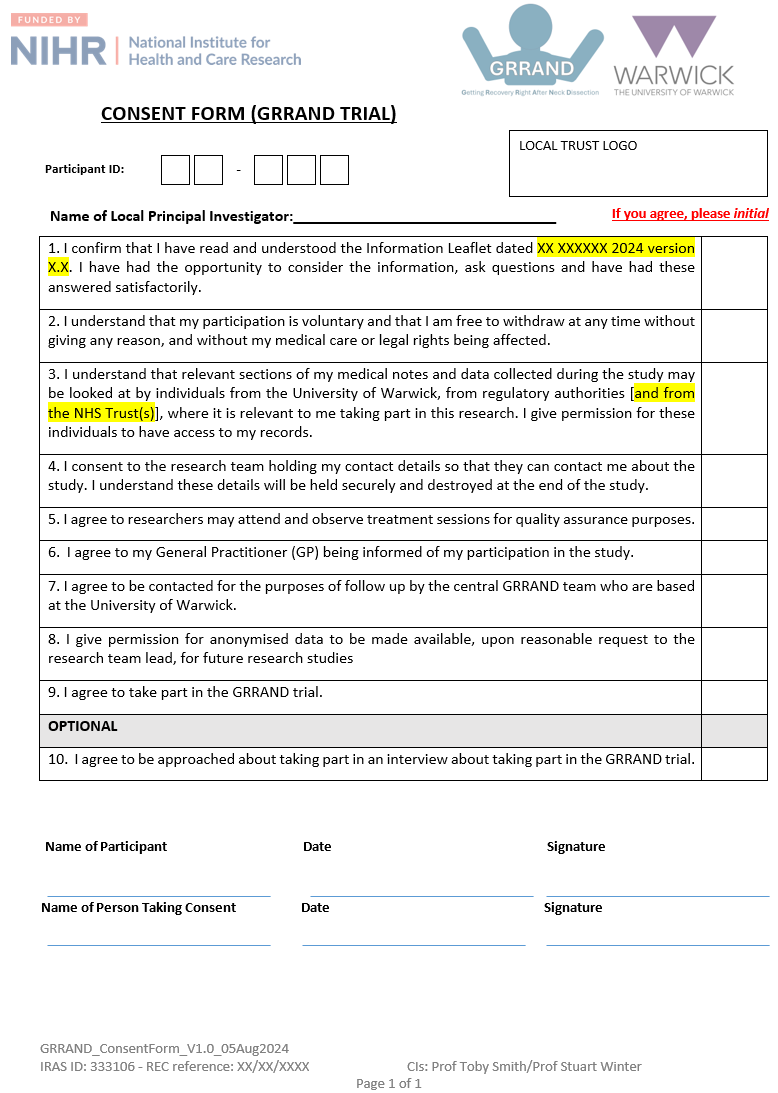


**Supplementary File 2:** A summary of core trial information presented in the WHO trial registration dataset format.

| **Data Category** | **Information** |
| --- | --- |
| **Primary Registry and Trial Identifying Number** | ISRCTN13855775 |
| **Date of Registration in Primary Registry** | 20 Dec 2024 |
| **Secondary Identifying Numbers** | NIHR158902; IRAS: 333106 |
| **Source(s) of Monetary or Material Support** | National Institute for Health Research, Health Technology Assessment |
| **Primary Sponsor** | University of Warwick |
| **Contact for Public Queries** | grrand@warwick.ac.uk |
| **Contact for Scientific Queries** | Professor Toby Smith (Co-CI), Warwick Clinical Trials Unit, University of Warwick or Professor Stuart Winter (Co-CI), Nuffield Department of Surgery, University of Oxford |
| **Public Title** | What is the clinical and cost-effectiveness of a personalised, physiotherapy-led, rehabilitation intervention (the GRRAND programme) compared to best practice NHS, post-discharge care for adults undergoing neck dissection for head and neck cancer? |
| **Scientific Title** | Getting Recovery Right After Neck Dissection for Head and Neck Cancer (GRRAND) |
| **Country of Recruitment** | UK |
| **Health Condition(s) or Problem(s) Studies** | Head and neck cancer |
| **Intervention(s)** | Experimental: Six (1-hour) sessions of a personalised physiotherapy-led rehabilitation programme (GRRAND programme) delivered over six months  Control: best practice NHS, post-discharge care (self-directed rehabilitation) |
| **Key Inclusion and Exclusion Criteria** | Inclusion criteria:   1. People aged 18 years or over 2. Diagnosis of HNC with requirement for a neck dissection as part of their treatment with curative intent. Including those undergoing completion neck dissection following positive sentinel node biopsy or open neck node biopsy. 3. Able to attend out-patient physiotherapy appointments. 4. Provide informed consent.   Exclusion criteria:   1. People for whom intensive post-discharge physiotherapy is expected (e.g., scapula/scapula tip and/or latissimus dorsi free flaps or components thereof). This constitutes 3-5% of the neck dissection population and clinical equipoise regarding the role of physiotherapy is less evident [20]. 2. People with a pre-existing, long-term disease affecting the shoulder, e.g., hemiplegia. 3. People who had prior neck dissection surgery on the affected side. 4. People undergoing only Lymph Node Biopsy or Sentinel Lymph Node Biopsy. 5. Previous entry in the present trial. 6. Unable to adhere to trial processes.   N.B Patients who have been excluded from the trial due to meeting Exclusion Criteria 4 may subsequently become eligible for inclusion IF they require complete neck dissection. |
| **Study Type** | Interventional  Allocation: randomised; individual assignment  Phase 3 |
| **Date of First Enrolment** | May 2025 |
| **Same Size** | 390 |
| **Recruitment Status** | Recruiting at time of submission |
| **Primary Outcome(s)** | Shoulder pain and function at 12-months using the participant-reported Shoulder Pain and Disability Index (SPADI) questionnaire (total score) |
| **Key Secondary Outcomes** | All assessed at six weeks, three, six and 12-months unless specified otherwise   - SPADI total score (secondary outcomes at six weeks and 6-months only) - Individual SPADI domains (pain and disability ) - Health-related quality of life (EQ-5D-5L*; EORTC cancer-specific questionnaires (C30(core); H&N35(head and neck specific)) - Mental wellbeing using the Short Warwick-Edinburgh Mental Wellbeing Scale - Exercise adherence using the Exercise Adherence Rating Scale (EARS) at six-weeks only - Adverse events including surgical complications* - Health resource use questionnaire*   (*three-month assessment for adverse events, EQ-5D-5L and health utilisation questionnaire only) |
| **Ethics Review** | London - Brent Research Ethics Committee; 15^th^ October 2024 |

| **Supplementary File 3:** Schedule of trial enrolment, interventions, and assessment.   \| **Visit/follow-up number** \| **-1** \| **1** \| **2** \| **3** \| **4** \| **5** \| **6** \| **7** \| \| --- \| --- \| --- \| --- \| --- \| --- \| --- \| --- \| --- \| \| **Visit/follow-up** \| **Screening** \| **Baseline** \| **In-Patient Pre-Discharge** \| **Intervention Period** \| **6 Weeks Post-Randomisation** \| **3 Months Post-Randomisation** \| **6 Months Post-Randomisation** \| **12* Months Post-Randomisation** \| \| **Time after randomisation (±window)** \| **-** \| **0** \| **Data to be collected pre-discharge, except for histology data which will be available within 4-6 weeks** \| **Data within 4 weeks of completion of allocated treatment** \| **(±2wks)** \| **(±1m)** \| **(±1m)** \| **(±2m)** \| \| Check eligibility ^$^ \| ✓ \|  \|  \|  \|  \|  \|  \|  \| \| Invitation to study ^$^ \| ✓ \|  \|  \|  \|  \|  \|  \|  \| \| Informed consent ^$^ \|  \| ✓ \|  \|  \|  \|  \|  \|  \| \| Medical history ^$^ \|  \| ✓ \|  \|  \|  \|  \|  \|  \| \| Inclusion/exclusion criteria ^$^ \|  \| ✓ \|  \|  \|  \|  \|  \|  \| \| Age (years) ^$^ \|  \| ✓ \|  \|  \|  \|  \|  \|  \| \| Gender ^$^ \|  \| ✓ \|  \|  \|  \|  \|  \|  \| \| Weight (kg)/(stone/lbs) ^$^ \|  \| ✓ \|  \|  \|  \|  \|  \|  \| \| Height (cm)/(ft/inches) ^$^ \|  \| ✓ \|  \|  \|  \|  \|  \|  \| \| Ethnicity ^$^ \|  \| ✓ \|  \|  \|  \|  \|  \|  \| \| Drinking status ^$^ \|  \| ✓ \|  \|  \|  \|  \|  \|  \| \| Smoking status ^$^ \|  \| ✓ \|  \|  \|  \|  \|  \|  \| \| Hand dominance ^$^ \|  \| ✓ \|  \|  \|  \|  \|  \|  \| \| List of medical co-morbidities ^$^ \|  \| ✓ \|  \|  \|  \|  \|  \|  \| \| Employment status and current occupation (when appropriate) * \|  \| ✓ \|  \|  \| ✓ \| ✓ \| ✓ \| ✓ \| \| Shoulder Pain and Disability Index (SPADI)* \|  \| ✓ \|  \|  \| ✓ \|  \| ✓ \| ✓ \| \| EQ-5D-5L* \|  \| ✓ \|  \|  \| ✓ \| ✓ \| ✓ \| ✓ \| \| EORTC QLQ-C30* \|  \| ✓ \|  \|  \| ✓ \|  \| ✓ \| ✓ \| \| EORTC QLQ-H&HN43* \|  \| ✓ \|  \|  \| ✓ \|  \| ✓ \| ✓ \| \| Short Warwick-Edinburgh Mental Wellbeing Scale* \|  \| ✓ \|  \|  \| ✓ \|  \| ✓ \| ✓ \| \| Health resource use questionnaire* \|  \| ✓ \|  \|  \| ✓ \| ✓ \| ✓ \| ✓ \| \| Exercise Adherence Rating Scale* \|  \|  \|  \| ✓^ǂ^ \| ✓ \|  \|  \|  \| \| ASA grade ^$^ \|  \|  \| ✓ \|  \|  \|  \|  \|  \| \| Pre-operative cancer head and neck treatment (chemo or radiotherapy) ^$^ \|  \|  \| ✓ \|  \|  \|  \|  \|  \| \| Complications, AE, SAE details of accident & emergency attendances and hospital admissions (and reasons) ^$^* \|  \|  \| ✓ \| ✓ \| ✓ \| ✓ \| ✓ \| ✓ \| \| Operation date ^$^ \|  \|  \| ✓ \|  \|  \|  \|  \|  \| \| Operative procedure (Level of ND) ^$^ \|  \|  \| ✓ \|  \|  \|  \|  \|  \| \| Early post-operative complications (Clavien-Dindo Classification) \|  \|  \| ✓ \|  \|  \|  \|  \|  \| \| Location of HNC ^$^ \|  \|  \| ✓ \|  \|  \|  \|  \|  \| \| Preservation or resection of accessory nerve ^$^ \|  \|  \| ✓ \|  \|  \|  \|  \|  \| \| Primary cancer site ^$^ \|  \|  \| ✓ \|  \|  \|  \|  \|  \| \| Stage of tumour^$^ \|  \|  \| ✓ \|  \|  \|  \|  \|  \| \| Type of tumour ^$^ \|  \|  \| ✓ \|  \|  \|  \|  \|  \| \| Neck nodal status ^$^ \|  \|  \| ✓ \|  \|  \|  \|  \|  \| \| Randomisation ^$^ \|  \|  \| ✓ \|  \|  \|  \|  \|  \| \| Chemotherapy and radiotherapy treatment provision ^$*^ \|  \|  \| ✓ \|  \| ✓ \| ✓ \| ✓ \| ✓ \| \| GRRAND intervention CRF (physiotherapist completed) ^$^ \|  \|  \| ✓ \| ✓ \|  \|  \|  \|  \| |
| --- | --- | --- | --- | --- | --- | --- | --- | --- | --- | --- | --- | --- | --- | --- | --- | --- | --- | --- | --- | --- | --- | --- | --- | --- | --- | --- | --- | --- | --- | --- | --- | --- | --- | --- | --- | --- | --- | --- | --- | --- | --- | --- | --- | --- | --- | --- | --- | --- | --- | --- | --- | --- | --- | --- | --- | --- | --- | --- | --- | --- | --- | --- | --- | --- | --- | --- | --- | --- | --- | --- | --- | --- | --- | --- | --- | --- | --- | --- | --- | --- | --- | --- | --- | --- | --- | --- | --- | --- | --- | --- | --- | --- | --- | --- | --- | --- | --- | --- | --- | --- | --- | --- | --- | --- | --- | --- | --- | --- | --- | --- | --- | --- | --- | --- | --- | --- | --- | --- | --- | --- | --- | --- | --- | --- | --- | --- | --- | --- | --- | --- | --- | --- | --- | --- | --- | --- | --- | --- | --- | --- | --- | --- | --- | --- | --- | --- | --- | --- | --- | --- | --- | --- | --- | --- | --- | --- | --- | --- | --- | --- | --- | --- | --- | --- | --- | --- | --- | --- | --- | --- | --- | --- | --- | --- | --- | --- | --- | --- | --- | --- | --- | --- | --- | --- | --- | --- | --- | --- | --- | --- | --- | --- | --- | --- | --- | --- | --- | --- | --- | --- | --- | --- | --- | --- | --- | --- | --- | --- | --- | --- | --- | --- | --- | --- | --- | --- | --- | --- | --- | --- | --- | --- | --- | --- | --- | --- | --- | --- | --- | --- | --- | --- | --- | --- | --- | --- | --- | --- | --- | --- | --- | --- | --- | --- | --- | --- | --- | --- | --- | --- | --- | --- | --- | --- | --- | --- | --- | --- | --- | --- | --- | --- | --- | --- | --- | --- | --- | --- | --- | --- | --- | --- | --- | --- | --- | --- | --- | --- | --- | --- | --- | --- | --- | --- | --- | --- | --- | --- | --- | --- | --- | --- | --- | --- | --- | --- | --- | --- | --- | --- | --- | --- | --- | --- | --- | --- | --- | --- | --- | --- | --- | --- | --- | --- | --- | --- | --- | --- | --- | --- | --- | --- | --- | --- | --- | --- | --- | --- | --- | --- | --- | --- | --- | --- | --- | --- | --- | --- | --- | --- | --- | --- | --- | --- | --- | --- | --- | --- | --- | --- | --- | --- | --- | --- | --- | --- | --- | --- | --- | --- |

^$^ site completed; ^*^ participant completed; ^ǂ^ GRRAND group only

**Supplementary File 4:** Template for Intervention Description and Replication (TIDieR) checklist for the GRRAND programme.

|  | | **Where Located** | |
| --- | --- | --- | --- |
| **Item number** | **Item** | **Primary paper (page number)** | **Other (details)** |
| 1 | **BRIEF NAME**  Provide the name or a phrase that describes the intervention | 5 | N/A |
| 2 | **WHY**  Describe any rationale, theory, or goal of the elements essential to the intervention | 1,6 | N/A |
| 3 | **WHAT**  Materials: describe any physical or informational materials used in the intervention, including those provided to participants or used in intervention delivery or in training providers. | 5-7 | N/A |
| 4 | Procedures: Describe each of the procedures, activities, and/or processes used in the intervention including any enabling or support activities. | 5-7 | N/A |
| 5 | **WHO PROVIDED**  For each category of intervention provider (e.g. psychologist, nursing assistant), describe their expertise, background and any specific training given. | 5 | N/A |
| 6 | **HOW**  Describe the modes of delivery (e.g. face-to-face or by some other mechanism, such as internet or telephone) of the intervention and whether it was provided individually or in a group | 5 | N/A |
| 7 | **WHERE**  Describe the type(s) of location(s) where the intervention occurred, including any necessary infrastructure or relevant features | 5 | N/A |
| 8 | **WHEN AND HOW MUCH**  Describe the number of times the intervention was delivered and over what period of time including the number of sessions, their schedule, and their duration, intensity or dose | 5,6 | N/A |
| 9 | **TAILORING**  If the intervention was planned to be personalised, titrated or adapted, then describe what, why when, and how | 6 | N/A |
| 10 | **MODIFICATIONS**  If the intervention was modified during the course of the study, describe the changes (what, why, when, and how) | 6 | N/A |
| 11 | **HOW WELL**  Planned: If intervention adherence or fidelity was assessed, describe how and by whom, and if any strategies were used to maintain or improve fidelity, describe them. | 3,10 | N/A |
| 12 | Actual: If intervention adherence or fidelity was assessed, describe the extent to which the intervention was delivered as planned. | N/A | N/A |
