# Supplementary material for: Getting recovery right after neck dissection for head and neck cancer (GRRAND): trial protocol for a multicentre, pragmatic randomised controlled trial with health economic evaluation and process evaluation
Source: BMJ Open. 2025 Oct 2;15(10):e109424. doi: 10.1136/bmjopen-2025-109424 (PMC12496047; doi:10.1136/bmjopen-2025-109424)
Supplement: Supplementary file 2 [file bmjopen-2025-109424.reviewer_comments.pdf]

PEER REVIEW HISTORY

BMJ Open publishes all reviews undertaken for accepted manuscripts. Reviewers are asked to complete a checklist review form and are provided with free text boxes to elaborate on their assessment. These free text comments are reproduced below.

ARTICLE DETAILS

Title (Provisional)

Getting Recovery Right After Neck Dissection for Head and Neck Cancer (GRRAND): trial protocol for a multi-centre, pragmatic randomised controlled trial with health economic evaluation and process evaluation.

Authors

Evans, Chrissy; Greene, Leanne; Hossain, Anower; Klear, Pritpal; Ratna, Mariam; Bradley, Helen; Lall, Ranjit; Mistry, Hema; Naghdi, Seyran; Reategui, Marco; Stevens, Tony; Bruce, Julie; Price, Ruth; Schache, Andrew; Dean, Sarah Gerard; Lamb, Sarah Elizabeth; Smith, Toby; Winter, Stuart C

This article was not externally reviewed at BMJ Open. Protocols that have been independently assessed prior to submission to BMJ Open are usually fast-tracked to publication on the grounds that further substantial changes will not be possible. This independent assessment will usually be external, independent review for both a substantial grant award from a non-commercial or government funder and ethics approval.
